# The Effect of Differential Attentional Focus Strategies on the Performance of Military Elite Shooters

**DOI:** 10.1155/2020/1067610

**Published:** 2020-11-25

**Authors:** Amin Amini, Mohammad Vaezmousavi

**Affiliations:** Department of Knowledge and Cognitive Intelligence, Imam Hossein University, Teheran, Iran

## Abstract

**Methods:**

This study is semiexperimental with an intragroup design. A number of 10 military marksmen (30-42 years old) with at least 10 years of experience in shooting performed under four attentional focus strategies in a counterbalanced design. In each strategy, two blocks (each consisting of 20 trials) were conducted. Shooters' performance was recorded using SCATT device and analyzed using the factorial variance analysis with repeated measure.

**Results:**

Results showed that the interactional effects of internal-external/relevant-irrelevant focuses of attention were significant on shooting record, shooting accumulation, and stability on the target center. Results suggest that the external-relevant attentional focus strategies were more effective than other focus strategies.

**Conclusion:**

The results of the study support the hypothesis that external-relevant attentional focus produced better scores, better accumulation, more stability at the target center, and less average fluctuation. Therefore, this attentional focus strategy improves performance precision of military elite shooters.

## 1. Introduction

Shooting activity requires consistency and accuracy [1]. Military and police personnel in peacekeeping roles are often required to make difficult and complex decisions regarding the use of deadly force in the midst of fluid, vague, and emotionally charged situations [2].

Various factors affect shooters' performance precision. Physiological factors, sensory factors (visual resolution and accuracy and the signal/noise ratio of the sensory channel), and motor factors such as recruitment pattern of muscles undoubtedly play roles in shooting performance [1]. Therefore, instructors use perceptual cognitive strategies, namely, attentional focus strategies to improve performance and its physiological outcomes [3].

Attentional focus influences muscle and nervous activities and ultimate performance outcomes [4]. Minor differences in the wording of focus instructions may bring different results. Research findings showed that when instructors change the words or phrases of the verbal instructions, the athlete's attention will be directed towards different aspects of the task and subsequently affect motor performance in a different way [5].

Attentional focus has been investigated from different perspectives and has been characterized as either associative (i.e., focusing on bodily sensation) or dissociative 7 (i.e., focusing on lyrics of a song that is being played) [5]. It has also been categorized as broad versus narrow, in terms of its width. Some other researchers have added more details to the attentional focus classification system [6]: adding dimensions of relevance (relevant versus irrelevant to the task) and direction with respect to the body of the performer (internal versus external) [7].

Although in their research, Beilock et al. and Castaneda and Gary emphasized the supremacy of the internal attentional focus [8–10], depending on the participants' skill level, there are considerable evidences indicating that directing attention externally to the effect of a movement in the environment improves performance [11].

The advantage of an external focus of attention over an internal focus has been well documented across a wide variety of skills. For example Zachry et al. showed that in basketball free throw, an external focus of attention produced better results comparing to an internal one [12]. Similar results were found by Wulf and Su and Bell and Hardy in golf shot accuracy [13, 14]. Marchant et al. in dart throwing skill [15] and Porter et al. in standing long jump [16] found similar results.

Wulf et al. outlined the mechanism for superiority of external attentional focus over internal attentional focus using constrained action hypothesis [17]. According to this hypothesis, an external focus of attention promotes a more automatic mode for motor control by unconscious, fast, and reflexive control processes, while an internal focus induces a conscious control, causing individuals to constrain their motor system by interfering with automatic control processes [17].

Among other mechanisms of evaluating the superiority of external attentional focus, Prinz's theory of common coding can be pointed out. According to this view, performance is more efficient when it is represented by the result of the motion, which is achievable by an external attentional focus [18]. In other words, perception and action can effectively communicate only if their focus refers to features of the external world. Accordingly, the common currency of representations involved in perception and action needs to be distal features [19].

In recent years, constraints-led perspective also explains the underlying mechanism of external attentional strategies [20]. Based on this approach, external attentional strategy improves dynamic self-organizing characteristics of motion in the motor system and subsequently improves motor performance by facilitating the fitting of environmental information [20].

Task-relevant attentional strategy refers to condition in which the performer deliberately focuses on task-relevant aspects such as movement of the opponent or the movement of the object. In contrast, in the task-irrelevant terms, performer considers task-irrelevant aspects of information such as the audience' noise. There are evidences indicating that elite performers display their best performance in the task-irrelevant attentional situation; in contrast, beginners do their best in task-relevant attentional circumstances. Explicit monitoring theory [21] states that the effectiveness of attentional strategies may differ in novices when compared to elite performers [8]. In other words, beginners tend to consciously control the motor skill, while elite performers tend to use the automatic motor control system [9].

Though most studies examined only one dimension of attentional strategies, few studies simultaneously considered both dimensions and investigated their interaction (interaction of internal-external attention strategies and task-relevant and irrelevant attention strategies). Castañeda and Gray concluded that batting performance for highly skilled players was best in the irrelevant/external condition and worst in the relevant/internal condition. Performance of less-skilled batters was significantly better in task-relevant conditions regardless of being external or internal [10]. Also, the results of a study by Russell et al. showed that an external, task-oriented focus of attention is needed to facilitate performance in novices [11].

It is often argued that an external focus of attention produces better outcomes because it results in better organization of the motor plan. For this to be true, it is important to show that the opposite focus of attention does not improve performance as much as an external focus actually does. On the other hand, we need to keep in mind that the explanations given by each line of research are mutually exclusive: results supporting one hypothesis will provide evidence against the other. In order to improve the understanding of how different attentional focus strategies operate in military shooting performance, researchers need to integrate these approaches to observe the effect of the interaction of these strategies on performance.

Therefore, the purpose of present study was to examine the effect of various attentional strategies (four attention conditions of external/relevant, external/irrelevant, internal/relevant, and internal/irrelevant) on precision performance of military elite shooters.

## 2. Methods and Materials

A number of 10 elite military shooters (all males, mean age: 36.8 years (SD: 8.6) and 9.8 years (SD: 7.13) of experience as a military shooter) volunteered to participate in this study. Due to the high professional standards in this skill, there were no more than 10 participants available for this study. All participants were trained in firearms safety and familiar with shooting a Kalashnikov AK-101 7.62 caliber. All participants self-reported their right hand as their dominant hand and had no history of physical or mental disabilities or injury. The University's Institutional Review Board approved the study, and all participants filled out consent forms.

**Figure 1:**
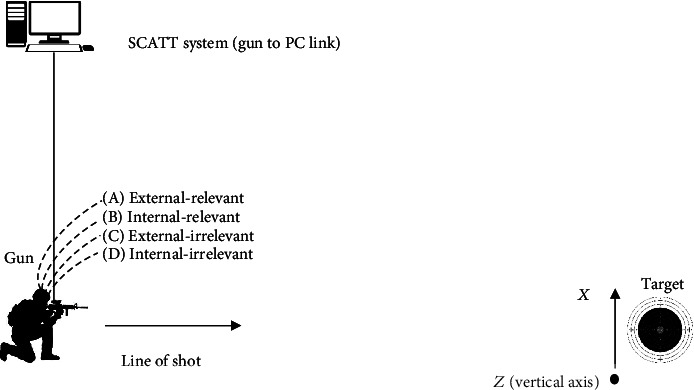
Testing setup.

The present study was conducted in an indoor shooting range with individual section lanes, with solitary fighting weapons used for every participant. Participants' shooting performance was tested using SCATT kit, on the same day. SCATT is a kit used to analyze shooting; it is comprised of a software (version 602) installed on a computer as well as hardware (an optical sensor fixed on the barrel, a target control unit, and an electronic target which can be installed at a distance of 4 to 12 meters from the shooter and target interface cable). This system displays the target image on the screen of the computer which simultaneously displays the trace of the point of aim by the optical sensor on the barrel on the screen. When the weapon trigger is activated, the point of impact is displayed on the screen. The sequence of the attentional conditions was the same for each participant ((A) external-relevant, (B) internal-relevant, (C) external-irrelevant, and (D) internal-irrelevant) (Figure 1).

The nomenclature presents the parameters used to indicate shooting performance and aim-point fluctuation (Table 1). All aim-point parameters relate to the last 1 second prior to the shot. All shooting performance and aim-point parameters were measured using a SCATT shooting training and analysis system, version 1.1 (Zao SCATT, Russia), similar to that used by Zatsiorsky and Aktov [22]. Briefly, this system employs a laser aligned with the gun barrel and an instrumented target to detect the position of the aim point on the target. The *X*-axis was aligned horizontally, and the *Z*-axis was aligned vertically on the target. The manufacturers quote the accuracy of this system as ±0.1 mm [23].

Shooting performance was indicated by score (out of 10.9), error in the horizontal (*X*) axis, and error in the vertical (*Z*) axis. These parameters are included in the nomenclature. The maximum score of 10.9 is consistent with the scoring protocol during finals in international shooting competitions, in which the point of shot on target is measured to one decimal place. Aim-point fluctuation parameters were quantified from 1 s to shot [24].

After providing the necessary explanation, participants filled in informed consent forms. Each participant was allowed to have 10 shots to fix the weapon (the score was not recorded). Then, in a within-group design, the participants took 80 counterbalanced trials in four conditions ((A) external-relevant, (B) internal-relevant, (C) external-irrelevant, and (D) internal-irrelevant). Therefore, each condition consisted of 20 shots. Participants took a period of 2-minute rest after 5 shots. In the internal-relevant condition, participants were required to mentally focus on the movement of their forearm, wrist, and fingers. In the external-relevant condition, they were asked to mentally focus on the target. In the internal-irrelevant condition, participants were required to mentally focus on their breathing rhythm and judge about the number of inhale and exhale in each trial. In the external-irrelevant condition, the participants had to mentally focus on an auditory stimulus which was simultaneously and randomly presented by the program designed in the Mat-lap software and then judge about the bass or treble nature of that sound. To improve the effectiveness of the attention strategies, the experimenter reminded the participants to focus according to the mentioned attentional strategy before and during the trials and do their best to obtain the highest shooting scores. Before each trial, the participants read aloud the attentional strategy instruction relevant to their states, written on a sheet of paper. This was to increase the effectiveness of the attentional strategies.

The Shapiro–Wilk test was used to assess the normality of data distribution for each variable. The Mauchly's test of sphericity was also used to test the assumption of sphericity, the Box's M test was used to test the homogeneity of covariance matrices, and eventually, the Levene test was used to assess the equality of variances. To data analysis, a 2 (external − internal focus) × 2 (relevant − irrelevant focus) repeated measure ANOVA was used. Data analysis was performed using the SPSS v. 20 software at a significance level equal or less than 0.05.

## 3. Ethics Approval and Consent to Participate

This study received ethical approval from the Institutional Review Board of the Baqiyatallah University of Medical Sciences (IR.BMSU.REC.1396.642), and written informed consent was obtained from all participants.

## 4. Results

The second table (Table 2) illustrates the mean and standard deviation of precision performance (record, accumulation, average fluctuations, horizontal fluctuations, vertical fluctuations, and stability at the target center) at differential attentional focus strategies.

The epsilon value was lying between 1/(4-1) and 1, where 4 is the number of levels ((1) external-relevant, (2) internal-relevant, (3) internal-irrelevant, and (4) external-irrelevant) on which our dependent variable differential attentional focus strategies have been measured.

The next table (Table 3) shows an overall significant difference between the means at the differential attentional focus strategies. This table demonstrates the *F* value for “2 (external − internal focus) × 2 (relevant − irrelevant focus)” factor, its associated significance level, and effect size (partial eta squared).

The main effect of internal-external attentional strategic conditions in precision performance was statistically significant. Considering the mean precision performance (record (*P* = 0.001), accumulation (*P* = 0.001), average fluctuations (*P* = 0.001), horizontal fluctuations (*P* = 0.001), vertical fluctuations (*P* = 0.001), and stability at the target center (*P* = 0.001)) of participants in any of the internal and external focus conditions, it can be concluded that the participants performed significantly better in the external-relevant and external-irrelevant attentional strategies comparing to internal-relevant and internal-irrelevant focus conditions.

The main effect of relevant-irrelevant focus was also statistically significant in precision performance (record (*P* = 0.005), accumulation (*P* = 0.001), average fluctuations (*P* = 0.001), and stability at the target center (*P* = 0.001)), and the participants performed better in the relevant focus conditions rather than relevant focus.

The interaction effect of relevant-irrelevant and internal-external attentional strategies in precision performance (record (*P* = 0.001), accumulation (*P* = 0.001), average fluctuations (*P* = 0.001), vertical fluctuations (*P* = 0.002), and stability at the target center (*P* = 0.001)) was also significant (Table 3).

**Figure 2:**
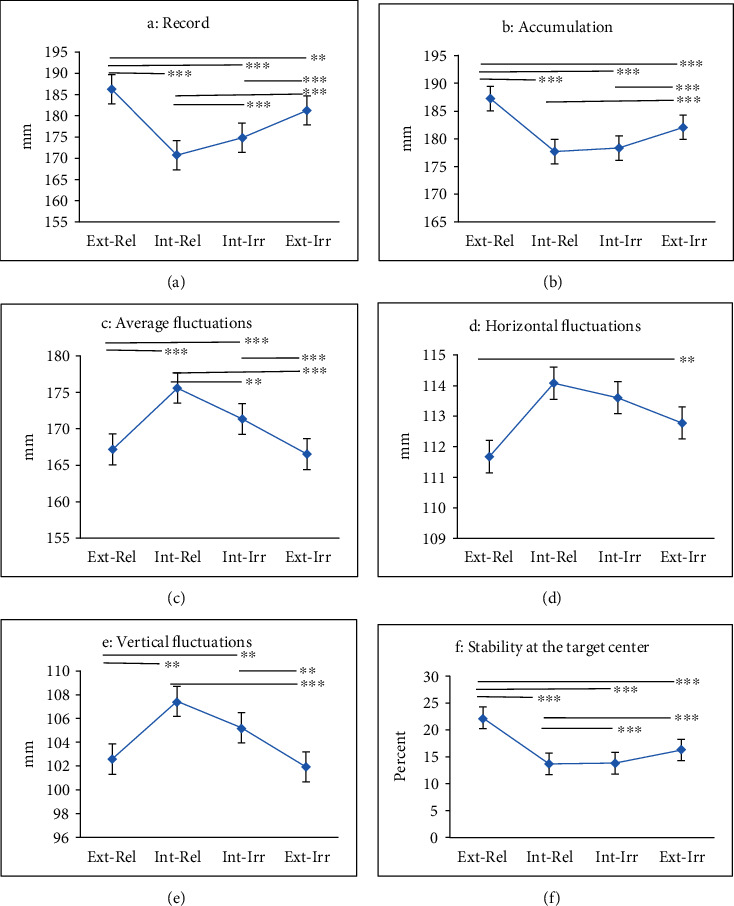
The effects of four types of attentional focus strategies [(1) external-relevant, (2) internal-relevant, (3) internal-irrelevant, and (4) external-irrelevant] on some performance indicators of military elite shooters [(a) record, (b) accumulation, (c) average fluctuations, (d) horizontal fluctuations, (e) vertical fluctuations, and (f) stability at the target center]. There is a significant difference in external-relevant strategy performance to other strategies in the three indexes record, accumulation, and stability at the target center (^∗∗∗^Significance level *P* ≤ 0.001).

Figures 2(a) and 2(f) show the average scores of different attentional focus strategies of research for different attentional focus strategies.

## 5. Discussion

This study examined the effect of different attentional focus strategies on performance of military elite shooters. Two types of attentional focus strategies were employed: “internal/external” and “task-relevant/task irrelevant.” The general hypothesis was “the interaction of various attentional strategies would significantly affect performance”; and the external-relevant focus of attention would produce the best results.

The results of this experiment showed that external-relevant focus of attention produced better scores, better accumulation, more stability at the target center, and less average fluctuation. Therefore, the results of the study retain the hypothesis of the study and indicate that external-relevant attentional focus improves performance precision of military elite shooters [11]. Although this research paradigm had not been previously carried out on military shooting performance, the overall results are in line with several previous findings which indicate the advantages of external-relevant focus of attention over other attentional strategies [8–10, 21].

The constrained action hypothesis suggests that the external focus of attention prevents from top-down constraints on the coordination of movement [5]; therefore, it results in superior motor performance compared to an internal focus of attention [25]. The facilitating effect of the external attentional focus has also been confirmed by the self-invoking trigger hypothesis in which an external focus of attention enhances movement effectiveness and efficiency [26].

The self-invoking trigger hypothesis was proposed by Wulf and Lewthwaite who suggested that an internal focus of attention triggers people to engage in self-evaluation and self-regulatory processes in an attempt to gain control over their thoughts and feelings. If the addition of these processes exceeds the attentional capacity, automatic control of the motor program may become disrupted and leads to declines in motor performance [26].

Also, results of the present study may well be explained by constrain-led perspective [27]. The participants who used external attentional focus have been able to well receive environmental affordances, provide themselves the necessary environmental information, and improve the dynamic self-organization characteristics of the motor system, as well as finally improve their performance precision. In other words, paying attention to the effects of moving in the environment gives direction to the searching processes for affordances related to the task execution and also directs executive system in self-organizing related constraints, and therefore improves task execution [20].

Among the various attentional strategies presented in this study, internal-relevant strategy presented the lowest score, lowest accumulation, lowest stability at the target, and the highest fluctuations, which is significantly inferior to the external-relevant attentional strategy. This is compatible with the proposed principles in the constraint action hypothesis which indicate that attempts to consciously control the movement in the form of the internal attentional focus confine the motor system, prevent processes that control the movement, and ultimately disturb automatic control of the motor system, therefore weaken the motor performance [28].

Based on what we have learnt from explicit processes theory [21], one may suggest that unlike beginners, elite participants show better performance in attentional strategies irrelevant to the tasks due to the lack of step-by-step assessment of motor skills. Since all participants of the present study were elite shooter, the explicit processes theory was also retained in the present study. Marchant et al. showed that for dart throwing, the compatibility with the external focus of attention (paying attention to the dart flight) was much more influential than with internal focus (paying attention to the hand) [29]. Findings of the present study are not compatible with the some previous research findings which reported an equal effect of adopting internal and external focus of attention among the elite participants [30].

Studying performance of the elite always has limitations. First, it is the limited number of participants. Authors were not able to induce voluntarily participation of any more elite military shooters; there is no need to mention that their total number is apparently limited too. This was somewhat justified by the limited sample size in previous similar studies [30, 31]. The second limitation is the absence of a baseline condition when comparing various attentional strategies, as experimental strategies were compared with each other. Although we provided explicit instructions on the focus of attention and supplemented the instructions with cueing throughout each set, there was not a clear way to make sure that subjects were actually focusing as directed. It remains possible that some subjects did not stick to the proper focus in at least some of the sets, which in turn may have altered the results. This is the third limitation of this study.

## 6. Conclusions

This study expanded the previous findings by demonstrating the greater effectiveness of external, relevant attentional strategy over other attentional strategies for elite shooting. Results of this study indicate the significance of using standard attentional strategies during training or assessing shooting skill. As the results show, changing attentional strategy leads to remarkable performance variations. Depending on the skill level of the participants, these strategies could differ. In general, given the importance and necessity of considering perceptive-cognitive skills in shooting, especially at the military level, findings of this study suggest that employing external attentional strategy, yet irrelevant to the task, would lead to improvement in the performance. Therefore, along with related hypothesis and theories in the field, findings of the present study suggest instructors to direct elite performers' attention to the external, yet irrelevant cues to the task.

## Figures and Tables

**Table 1:** The parameters used to indicate shooting performance and aim-point fluctuation.

Nomenclature	
Parameter	Definition
*Performance (SCATT)*	
Record/score	Out of a possible 10.9; high record equals performance improvement
Accumulation	Horizontal distance (mm) of shot from target center and vertical distance (mm) of shot from target center; higher accumulation means better performance
*Aim-point fluctuation (SCATT)*	
Average fluctuations	% time, the aim point spends in the 10-scoring zone, used to indicate the accuracy of aiming; lower average fluctuations mean better performance
Stability at the target center	% time, the aiming point spends in an area of the size of the 10-scoring zone (maximum), used to indicate the steadiness of aiming; higher stability at the target center means better performance
Horizontal fluctuations	Total distance (mm), the aim-point trace moves in the *X* (horizontal) axis; lower horizontal fluctuations mean better performance
Vertical fluctuations	Total distance (mm), the aim-point trace moves in the *Z* (vertical) axis; lower vertical fluctuations mean better performance

**Table 2:** Means and standard deviations for the differential attentional focus strategies as a performance precision of military elite shooters.

	Record	Accumulation	Average fluctuations	Horizontal fluctuations	Vertical fluctuations	Stability at the target center
*External-relevant*						
M	186.28	187.25	167.21	111.67	102.60	22.31
SD	3.33	1.47	1.09	1.44	0.91	1.32
*Internal-relevant*						
M	170.74	177.70	175.64	114.07	107.48	13.74
SD	1.87	1.39	3.19	1.63	1.29	0.97
*Internal-irrelevant*						
M	174.79	178.35	171.40	113.60	105.24	13.87
SD	1.95	1.52	3.45	1.63	1.29	0.89
*External-irrelevant*						
M	181.30	182.07	166.54	112.77	101.96	16.32
SD	0.67	0.95	1.48	1.43	0.90	1.08

**Table 3:** Results of factorial [2 (external − internal focus) × 2 (relevant − irrelevant focus)] analysis of variance repeated measures (ANOVA-RM) for performance precision of military elite shooters in differential attentional focus strategies.

	Type III sum of squares	df	*F*	Sig.	Partial eta squared
*Record*					
External-internal	1133.160	1	263.885	0.001^∗^	0.880
Relevant-irrelevant	217.832	1	61.824	0.005^∗^	0.648
External-internal ^∗^relevant-irrelevant	205.662	1	47.894	0.001^∗^	0.571
*Accumulation*					
External-internal	440.232	1	159.841	0.001^∗^	0.816
Relevant-irrelevant	82.080	1	29.803	0.001^∗^	0.453
External-internal ^∗^relevant-irrelevant	44.732	1	16.242	0.001^∗^	0.311
*Average fluctuations*					
External-internal	408.960	1	102.943	0.001^∗^	0.741
Relevant-irrelevant	73.170	1	18.418	0.001^∗^	0.338
External-internal ^∗^relevant-irrelevant	23.560	1	5.931	0.001^∗^	0.141
*Horizontal fluctuations*					
External-internal	26.082	1	13.113	0.001^∗^	0.267
Relevant-irrelevant	0.992	1	0.499	0.485	0.014
External-internal ^∗^relevant-irrelevant	6.162	1	3.098	0.087	0.079
*Vertical fluctuations*					
External-internal	166.464	1	89.760	0.001^∗^	0.714
Relevant-irrelevant	6.400	1	3.451	0.071	0.087
External-internal ^∗^relevant-irrelevant	20.736	1	11.181	0.002^∗^	0.237
*Stability at the target center*					
External-internal	93.636	1	77.049	0.001^∗^	0.682
Relevant-irrelevant	303.601	1	249.820	0.001^∗^	0.874
External-internal ^∗^relevant-irrelevant	85.849	1	70.641	0.001^∗^	0.662

^∗^Significance level *P* ≤ 0.05.

## Data Availability

The measured data used to support the findings of this study are available from the corresponding author upon request.
